# 

**Published:** 1914-04

**Authors:** 


					﻿■ OFFICIAL ORGAN—State Society Social Hygiene and Eugenics.
Vol. XXIX
APRIL; 1914
No. 10
The "Red Back"
TEXAS MEDICAL JOLxNAL
ESTABLISHED JULY, 1885
PUBLISHED MONTHLY* AT AUSTIN, TEXAS, BY
^RIN’f'EO^BY THE VON BOECKMANN-JONES L)' ,
Subscription, $1.00 a Year, In Advance. -	-	<n <'Cnts
Entered at the Postoffice at Austin, Texas, as second class mail matter.
a
I
?■
E
H
I
Z
>
f-
z
i
fi
				

